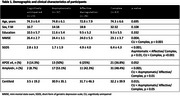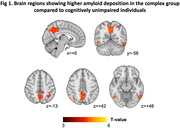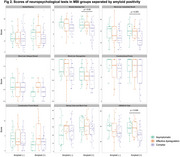# Associations between mild behavioral impairment and amyloid deposition in amnestic mild cognitive impairment

**DOI:** 10.1002/alz70857_100594

**Published:** 2025-12-24

**Authors:** Eun Jin Yoon, Heejung Kim, Jun‐Young Lee, Sang Jeong Kim, Yu Kyeong Kim

**Affiliations:** ^1^ Seoul National University, Seoul, Korea, Republic of (South); ^2^ SMG‐SNU Boramae Medical Center, Seoul, Korea, Republic of (South); ^3^ Seoul National University College of Medicine, Seoul, Korea, Republic of (South)

## Abstract

**Background:**

In our previous work, we found that amnestic mild cognitive impairment (aMCI) patients with multiple mild behavioral impairment (MBI) domain symptoms (complex group) revealed a higher risk of progression to Alzheimer's disease compared with those with no MBI symptoms (asymptomatic group) or those with only affective dysregulation (affective dysregulation group). Here, we investigated whether the MBI subgroups associated with early amyloid deposition in aMCI individuals.

**Method:**

A total 121 older adults with aMCI and 23 cognitively unimpaired (CU) individuals underwent ^18^F‐florbetaben (FBB) PET scans. MBI was approximated using a transformation algorithm for the neuropsychiatric inventory and participants with aMCI were classified into asymptomatic, affective dysregulation, and complex groups. PET images were corrected partial volume effect (PVE) using modified Müller‐Gärtner method. Regional standardized uptake value ratio (SUVR) maps of FBB PET were calculated by dividing the mean of the cerebellar gray matter value. Voxel‐wise comparisons of FBB SUVR maps between groups were performed using general linear model after controlling for age and sex (*p* < 0.05, FWE corrected; k=100). Amyloidpositivity was defined as Centiloid > 21 using the PET images without PVE correction.

**Result:**

The 3 MBI groups of aMCI individuals showed higher prevalence of amyloid‐positive and higher values of Centiloid compared to CU individuals, however the global amyloid deposition did not differ between MBI groups in (Table 1). In voxel‐wise group comparisons, the complex group revealed higher FBB SUVRs in the bilateral precuneus, inferior temporal and angular cortices compared to CU individuals (Figure 1). The asymptomatic and affective dysregulation groups did not reveal the significant differences with CU individuals in the same statistical level. There were also no regional differences in FBB SUVRs between MBI groups. Among the amyloid‐positive patients, the complex group revealed relative lower cognitive scores compared to asymptomatic group, but the differences in cognitive function were not found in the amyloid‐negative patients (Figure 2).

**Conclusion:**

The multiple co‐occuring MBI domain symptoms in aMCI individuals were associated with higher regional amyloid deposition and severer cognitive impairment, suggesting more advanced disease stage. Therefore, evaluation of MBI could be useful for risk stratification in MCI individuals.